# Relative Contributions of All-*Trans* and 11-*Cis* Retinal to Formation of Lipofuscin and A2E Accumulating in Mouse Retinal Pigment Epithelium

**DOI:** 10.1167/iovs.62.2.1

**Published:** 2021-02-01

**Authors:** Nicholas P. Boyer, Debra A. Thompson, Yiannis Koutalos

**Affiliations:** 1 Department of Ophthalmology, Medical University of South Carolina, Charleston, South Carolina, United States; 2 Department of Ophthalmology, University of Michigan School of Medicine, Ann Arbor, Michigan, United States; 3 Department of Biological Chemistry, University of Michigan School of Medicine, Ann Arbor, Michigan, United States

**Keywords:** lipofuscin, retinaldehyde, retinol dehydrogenase

## Abstract

**Purpose:**

*Bis*-retinoids are a major component of lipofuscin that accumulates in the retinal pigment epithelium (RPE) in aging and age-related macular degeneration (AMD). Although *bis*-retinoids are known to originate from retinaldehydes required for the light response of photoreceptor cells, the relative contributions of the chromophore, 11-*cis* retinal, and photoisomerization product, all-*trans* retinal, are unknown. In photoreceptor outer segments, all-*trans* retinal, but not 11-*cis* retinal, is reduced by retinol dehydrogenase 8 (RDH8). Using *Rdh8*^−/−^ mice, we evaluated the contribution of increased all-*trans* retinal to the formation and stability of RPE lipofuscin.

**Methods:**

*Rdh8^−^^/^^−^* mice were reared in cyclic-light or darkness for up to 6 months, with selected light-reared cohorts switched to dark-rearing for the final 1 to 8 weeks. The *bis*-retinoid A2E was measured from chloroform-methanol extracts of RPE-choroid using HPLC-UV/VIS spectroscopy. Lipofuscin fluorescence was measured from whole flattened eyecups (excitation, 488 nm; emission, 565–725 nm).

**Results:**

Cyclic-light-reared *Rdh8*^−/−^ mice accumulated A2E and RPE lipofuscin approximately 1.5 times and approximately 2 times faster, respectively, than dark-reared mice. Moving *Rdh8*^−/−^ mice from cyclic-light to darkness resulted in A2E levels less than expected to have accumulated before the move.

**Conclusions:**

Our findings establish that elevated levels of all-*trans* retinal present in cyclic-light-reared *Rdh8*^−/−^ mice, which remain low in wild-type mice, contribute only modestly to RPE lipofuscin formation and accumulation. Furthermore, decreases in A2E levels occurring after moving cyclic-light-reared *Rdh8*^−/−^ mice to darkness are consistent with processing of A2E within the RPE and the existence of a mechanism that could be a therapeutic target for controlling A2E cytotoxicity.

Lipofuscin is a fluorescent mixture of partially digested lipids and proteins that accumulates in the lysosomal compartment of post-mitotic cells,^1^^,^^2^ including the retinal pigment epithelium (RPE) in the eye.^3^ In the case of the RPE, the bulk of lipofuscin originates from material taken into the cells by daily phagocytic uptake of shed rod photoreceptor outer segments.^4^^,^^5^ Although its overall composition is unknown, RPE lipofuscin is known to contain several species of *bis*-retinoids,^6^ which are condensation products derived from vitamin A. RPE lipofuscin is potentially a factor in the pathogenesis of retinal disease, as it can act as a photosensitizer to mediate light-induced damage.^7^^–^^9^ In addition, the best-characterized *bis*-retinoid component of lipofuscin (A2E)^10^^,^^11^ displays a range of cytotoxic properties, including inhibition of lysosomal lipid processing^12^ and mitochondrial function.^13^ Thus, the pathways that generate lipofuscin and its *bis*-retinoid components have been the focus of significant research efforts, and the therapeutic effectiveness of suppressing *bis*-retinoid formation has been tested in clinical trials.^14^^,^^15^ The goal of the present work is to further define the mechanisms involved in *bis*-retinoid accumulation and processing in the RPE, in order to identify potential targets for future therapeutic efforts.

*Bis*-retinoids can form from reactions of 11-*cis* and all-*trans* isomers of retinal, the aldehyde form of vitamin A, with rod photoreceptor outer segment components.^16^^–^^18^ The 11-*cis* retinal is the light-sensitive chromophore of the rod photoreceptor photopigment rhodopsin^19^^,^^20^ and is generated in the RPE by the enzyme RPE65 using all-*trans* retinyl esters as substrate.^21^^–^^23^ The 11-*cis* retinal is transported to the rod photoreceptor outer segments where it combines with the apo-protein opsin to form rhodopsin.^24^ Light-induced photoisomerization of 11-*cis* retinal to all-*trans* retinal generates photoactivated rhodopsin, which initiates a cascade of reactions that culminate in a change in photoreceptor cell membrane potential leading to the light response.^19^^,^^25^ Subsequently, all-*trans* retinal is released from the photoactivated rhodopsin, and reduced to all-*trans* retinol by the rod outer segment enzyme retinol dehydrogenase RDH8^26^^–^^28^ using nicotinamide adenine dinucleotide phosphate (NADPH) as a cofactor.^29^ The narrow substrate specificity of RDH8^30^ enables efficient elimination of all-*trans* retinal while sparing the 11-*cis* retinal chromophore.

Most of RPE lipofuscin fluorescence is due to *bis*-retinoids, as evidenced by the low levels of RPE fluorescence in mice that lack the RPE65 enzyme and are unable to generate 11-*cis* retinal.^16^^,^^31^ Although both 11-*cis* and all-*trans* retinal can generate *bis*-retinoids in vitro, cyclic-light- and dark-reared wild-type mice show broadly similar levels of RPE lipofuscin and A2E accumulation,^16^ suggesting that most *bis*-retinoids originate from 11-*cis* retinal. The relative contributions of 11-*cis* and all-*trans* retinal to *bis*-retinoid formation are an important consideration needed to understand the mechanism of lipofuscin formation in the RPE and the therapeutic possibilities for controlling its accumulation.

In the present study, we have directly probed the extent of all-*trans* retinal contribution to RPE lipofuscin and A2E formation using mice that lack the RDH8 enzyme. Previous work has shown that, following light stimulation, the removal of all-*trans* retinal in the retinas of *Rdh8^−^^/^^−^* mice is markedly slowed compared to wild-type mice.^27^ Thus, in *Rdh8*^−/−^ mice, the potential contribution of elevated all-*trans* retinal to *bis*-retinoid formation can be maximized. We find that cyclic-light-reared *Rdh8^−^^/^^−^* mice accumulate RPE lipofuscin and A2E faster than dark-reared *Rdh8^−^^/^^−^* mice, but not at rates that would explain the accumulation achieved in the wild-type retinas in which the ratio of 11-*cis* to all-*trans* retinal is much higher. This leads us to conclude that accumulation of RPE lipofuscin and A2E under normal physiological conditions originates mainly from 11-*cis* retinal. We also find evidence for A2E processing within RPE cells, pointing to the existence of a mechanism that could be targeted to limit *bis*-retinoid accumulation.

## Methods

The original breeding pairs of *Rdh8^−^^/−^* mice were a generous gift of Dr. Krzysztof Palczewski. The mice were pigmented and had the Rpe65-Met450 variant.^32^ Animals were reared in cyclic light with a 12-hour light cycle (06:00–18:00), with the light intensity at cage level during the light part of the cycle being 130 to 170 lux; animals were also born and reared in the dark, in ventilated cabinets, and were exposed to dim red light only when checking on their health and for cage changes. Some animals were switched from light to dark-rearing for periods ranging from 1 to 8 weeks. Outcomes were evaluated in animals that were 1 to 6 months old. All animal procedures were carried out in accordance with protocols approved by the Institutional Animal Care and Use Committee of the Medical University of South Carolina; the authors adhere to the ARVO Statement for the Use of Animals in Ophthalmic and Vision Research.

Procedures for RPE and retina dissection, flat-mounting for RPE imaging, color photography, RPE fluorescence measurements, and A2E quantification followed exactly those described previously.^16^^,^^33^ Briefly, eyes were enucleated, and then hemisected at the level of the ora serrata in a mammalian physiological solution (in mmol/L: 130 NaCl, 5 KCl, 0.5 MgCl_2_, 2 CaCl_2_, 25 hemisodium-HEPES, 5 glucose, pH = 7.40). Lipofuscin fluorescence was measured from whole flattened eyecups with a 10 × lens (numerical aperture NA = 0.3) on an SP2 Leica Laser Scanning Confocal microscope (excitation, 488 nm; emission, 565–725 nm) as described.^16^ The total fluorescence from each eyecup was converted to “bead units” (BU), by measuring the fluorescence of intensity calibration beads (InSpeckOrange [540/560] Microscope Image Calibration Kit beads; Thermo Fisher Scientific, Waltham, MA, USA) under exactly the same image acquisition settings as for eyecups. Eyecup lipofuscin fluorescence is reported as “bead units per megapixel” (BU/MP), with one megapixel being equal to 2.44 mm^2^. For each age and condition, three animals providing six eyecups were used. Lipofuscin granule fluorescence emission spectra were measured from the same eyecups and on the same microscope with a 63 × oil-immersion objective (NA = 1.4). Color photographs (excitation, 450–490 nm; emission >510 nm) were taken on a Zeiss Axioplan 2 microscope (Carl Zeiss, Thornwood, NY, USA) using a 63 × oil immersion objective (NA = 1.4) with a Nikon D200 (Nikon, Inc., Melville, NY, USA) digital camera. The *bis*-retinoid A2E was measured from chloroform-methanol extracts of RPE-choroid samples with HPLC-UV/VIS spectroscopy.^16^ The segment of the chromatogram we used for measuring A2E levels does not contain a single peak. There may be additional components within the chloroform-methanol extract that co-elute and share a similar absorbance with A2E.^34^ We have previously established with liquid-chromatography mass spectrometry (LC-MS) that this fraction contains A2E, which makes about 50% of what is measured from the area under the curve (AUC) from the chromatogram.^34^ For experiments with mice reared in cyclic light, 6 eyecups were used for each experiment; with mice reared in darkness, 6 eyecups were used for each experiment for 1-month and 2-month old mice, and 4 eyecups were used for 3-month and 6-month old mice; with mice switched from light to dark-rearing, 2 eyecups were used for each experiment. Each experiment was repeated three times. All procedures were carried out under infrared or dim red light. All reagents were of analytical grade.

Error bars represent SEM. Statistical significance for the age-dependent increase of lipofuscin and A2E accumulation was tested with regression analysis; for individual comparisons, the *t*-test was used.

## Results

### Lipofuscin Granules in the RPE of *Rdh8^−^^/^^−^* Mice

**Figure 1.**
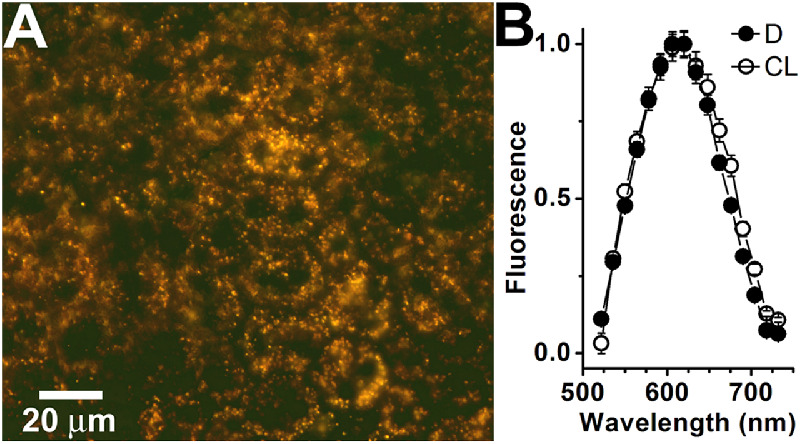
Lipofuscin accumulates in the RPE of *Rdh8^−/−^* mice. (**A**) True color fluorescence micrograph (excitation: 450–490 nm) of a flat-mounted RPE from 3-month-old dark-reared *Rdh8^−/−^* mouse. (**B**) Fluorescence emission spectra (excitation: 488 nm) of RPE lipofuscin granules (*n* = 101 for each) from 3-month-old cyclic-light- (CL, ○) and dark-reared (D, ●) *Rdh8^−/−^* mice. Error bars represent SEM.

Fluorescence photographs (excitation 450–490 nm) of RPE flatmounts from *Rdh8^−^^/^^−^* mice show the characteristic golden-orange granules of lipofuscin (Fig. 1A), similar to those found in wild-type mice.^16^ These granules are present in the RPE of both cyclic-light- and dark-reared *Rdh8^−^^/^^−^* mice, and exhibit their characteristic fluorescence emission spectra (excitation 488 nm) peak approximately 610 nm (Fig. 1B), independent of light- or dark-rearing or age (up to 6 months).

### RPE Lipofuscin Fluorescence and A2E Levels Increase Faster with Age in Cyclic-Light-Reared Than in Dark-Reared *Rdh8^−^^/^^−^* Mice

**Figure 2.**
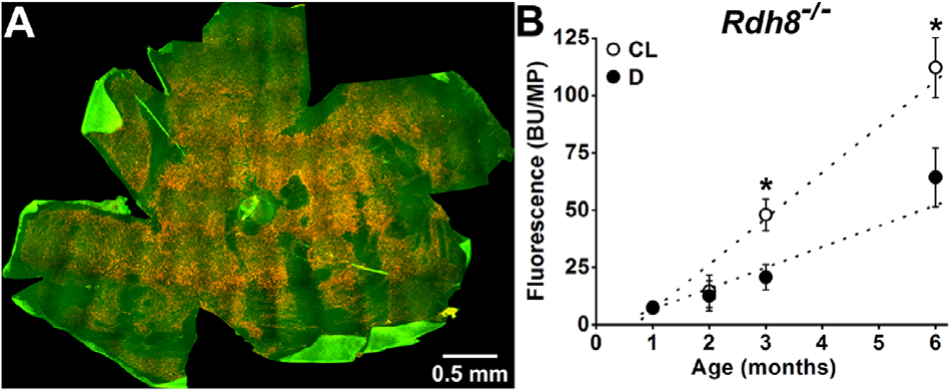
Total RPE fluorescence increases faster with age in cyclic-light-reared compared to dark-reared *Rdh8^−^^/^^−^* mice. (**A**) True color fluorescence micrograph (excitation: 450–490 nm) of a whole flat-mounted RPE of a 3-month-old cyclic-light-reared *Rdh8^−^^/^^−^* mouse. (**B**) Total RPE fluorescence (excitation: 488 nm; emission: 565–725 nm; measured in bead units per megapixel) increases with age in cyclic-light- (CL, ○) and dark-reared (D, ●) *Rdh8^−^^/^^−^* mice. *Straight lines* are regression lines with slopes of 20 BU/MP/month for cyclic-light-reared and 9.1 BU/MP/month for dark-reared mice. All experiments were conducted in triplicate. Error bars represent SEM. *Asterisks* denote statistical significance.

RPE lipofuscin levels were measured by collecting the fluorescence using an SP2 Leica Laser Scanning Confocal microscope (excitation 488 nm; emission 565–725 nm) from a whole flat-mounted eyecup. Figure 2A shows a fluorescence photograph (excitation 450–490 nm) of such a flat-mounted eyecup from a 3-month-old cyclic-light-reared *Rdh8^−^^/^^−^* mouse, obtained using an epifluorescence Zeiss Axioplan 2 microscope. In dark-reared *Rdh8^−^^/^^−^* animals, eyecup fluorescence was 7.5 ± 2.4 BU/MP at 1 month of age; it increased with age (*P* = 0.04) at a rate of 9.1 ± 1.9 BU/MP/month, reaching 64.3 ± 12.9 BU/MP at 6 months (Fig. 2B). In cyclic-light-reared animals, eyecup fluorescence was 7.3 ± 2.2 BU/MP at 1 month of age; it increased with age (*P* = 0.02) at a rate of 20.0 ± 2.7 BU/MP/month, and reaching 112.2 ± 13.1 BU/MP at 6 months (see Fig. 2B). Eyecup fluorescence was significantly higher in cyclic-light- compared to dark-reared animals at 3 and 6 months of age (one-tailed *t*-test, *P* = 0.006 and 0.01, respectively).

**Figure 3.**
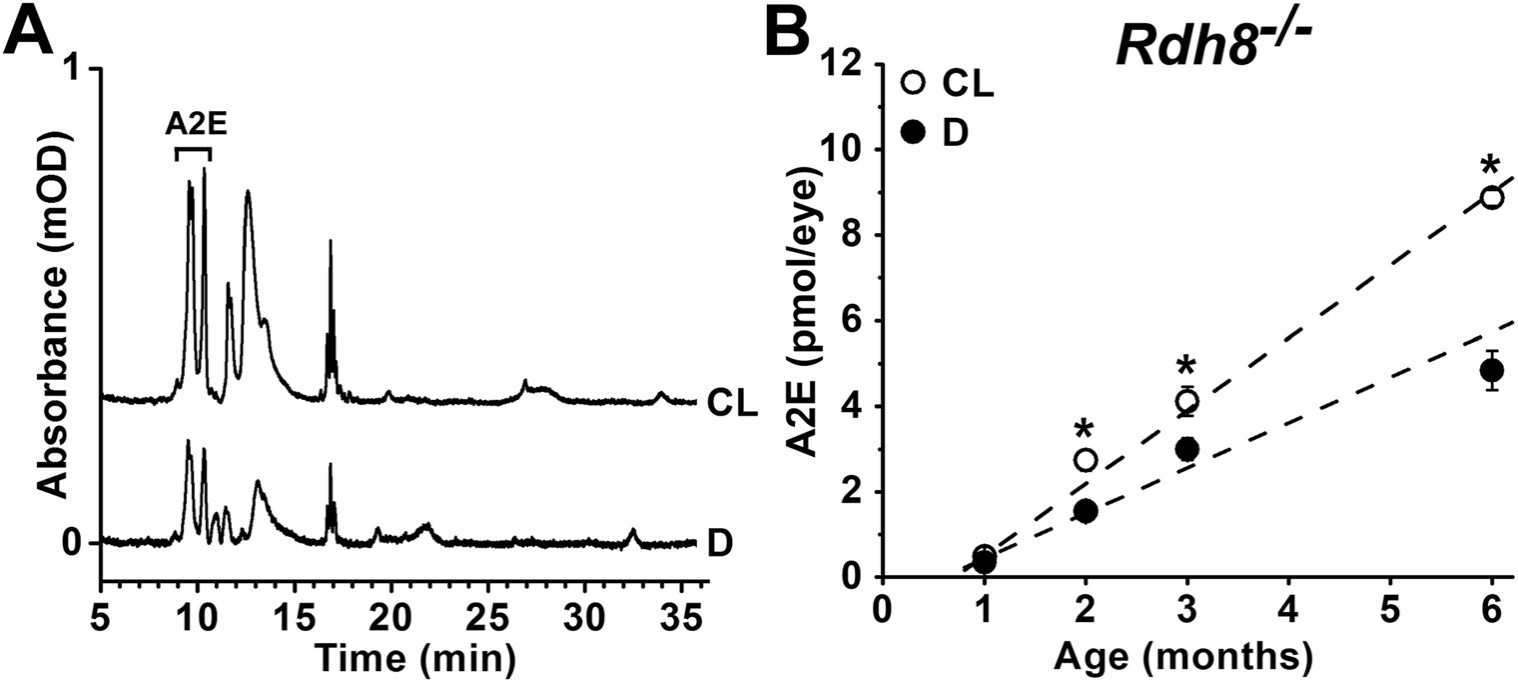
The RPE levels of the *bis*-retinoid A2E increase faster with age in cyclic-light-reared compared to dark-reared *Rdh8^−^^/^^−^* mice. (**A**) Chromatograms at 430 nm of RPE-choroid extracts from 6-month-old cyclic-light- (CL) and dark-reared (D) *Rdh8^−^^/^^−^* mice. Trace absorbance has been normalized to the number of eyes used, so it represents amount per eye. (**B**) RPE levels of A2E increase with age in cyclic-light- (CL, ○) and dark-reared (D, ●) *Rdh8^−^^/^^−^* mice. *Straight lines* are regression lines with slopes of 1.7 pmol/eye/month for cyclic-light-reared and 1.1 pmol/eye/month for dark-reared mice. All experiments were conducted in triplicate. Error bars represent SEM. *Asterisks* denote statistical significance.

Fluorescence intensity does not in general provide a direct measure of the mass of fluorescent material. In the case of lipofuscin, its overall composition is unknown, with the known components being the *bis*-retinoids. Among *bis*-retinoids, A2E constitutes the best characterized component and has commonly been used to ascertain whether increases in lipofuscin fluorescence are also associated with increases in the mass of a chemically identified component. A2E levels were measured by HPLC analysis of organic extracts of RPE-choroid tissue (Fig. 3A). Extracts from cyclic-light-reared animals contained larger amounts of A2E. In dark-reared *Rdh8*^−/−^ mice, the amount of A2E increased from 0.35 ± 0.1 pmol/eye at 1 month of age to 4.8 ± 0.5 pmol/eye at 6 months of age; its levels increased with age (*P* = 0.02), at a rate of 1.1 ± 0.2 pmol/eye/month (Fig. 3B). In cyclic-light-reared *Rdh8*^−/−^ mice, the amount of A2E increased from 0.49 ± 0.01 pmol/eye at 1 month of age to 8.9 ± 0.2 pmol/eye at 6 months of age; its levels increased with age (*P* = 0.001), at a rate of 1.7 ± 0.1 pmol/eye/month (see Fig. 3B). A2E levels were significantly higher in cyclic-light- compared to dark-reared animals at 2, 3, and 6 months of age (one-tailed *t*-test, *P* = 0.005, *P* = 0.03, and *P* = 0.0003, respectively).

In contrast to the RPE, there were no detectable differences in the A2E levels in the retina between cyclic-light- and dark-reared animals; at 6 months of age, levels were 1.9 ± 0.4 and 1.7 ± 0.1 pmol/eye, respectively (*P* = 0.65).

### Changes in A2E Levels in *Rdh8^−^^/^^−^* Mice Moved from Cyclic Light to Darkness

**Figure 4.**
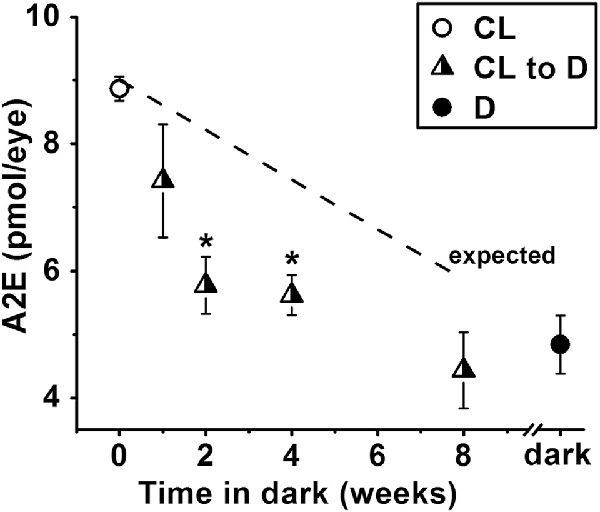
Moving *Rdh8^−^^/^^−^* mice from cyclic light to darkness decreases the accumulation of A2E in the RPE. Animals were moved to dark for different lengths of time before becoming 6 months old. A2E levels were measured at 6 months. All experiments were conducted in triplicate. The *straight line* shows the expected levels of A2E accumulation at the time the animals were moved to darkness. The cyclic light (CL, ○) and dark (D, ●) data points are from the experiments in Figure 3B. Error bars represent SEM. *Asterisks* denote statistical significance.

The significantly higher levels of A2E in the RPE of cyclic-light-reared versus dark-reared *Rdh8^−^^/^^−^* mice allowed us to examine whether moving animals from light to darkness affected A2E accumulation. Animals were initially reared in cyclic light and then moved to darkness for periods from 1 to 8 weeks, until they were 6 months old. At 6 months of age, mice that had spent the previous 8 weeks in darkness had the same levels of A2E as mice that were dark-reared for the entire 6-month period (Fig. 4). The simple linear time course of A2E accumulation in cyclic light (see Fig. 3B) allowed the estimation of the expected levels of A2E at the time points when the animals were moved to darkness (see Fig. 4, line labeled “expected”). For mice that were 5 months old when they were moved to darkness, the expected A2E levels at that time were 7.4 pmol/eye; at 6 months of age, the A2E levels were found to be significantly lower 5.6 ± 0.3 pmol/eye (*t*-test, *P* = 0.03). For mice that were 5.5 months old when they were moved to darkness, the expected A2E levels at that time were 8.0 pmol/eye; at 6 months of age, the A2E levels were found to be significantly lower 5.8 ± 0.5 pmol/eye (*t*-test, *P* = 0.048; see Fig. 4).

## Discussion

The cytotoxicity of *bis*-retinoids such as A2E and their potential role in the pathogenesis of AMD have made the sources and pathways contributing to their accumulation in the RPE the subject of extensive research efforts, as therapeutic strategies rely on understanding them. For example, if the major source for *bis*-retinoids were all-*trans* retinal, limiting light exposure – through the use of dark glasses – could provide a means to check *bis*-retinoid accumulation; if, however, the major source was 11-*cis* retinal, suppressing the generation of 11-*cis* retinal itself might be required. In human clinical trials aiming to slow the progression of AMD, formation of 11-*cis* retinal was suppressed either by limiting the availability of vitamin A with fenretinide,^14^ or, in a more direct fashion, by inhibiting 11-*cis* retinal generation with emixustat.^15^ Several participants in the trials experienced significant adverse effects, such as impaired dark adaptation, an expected side effect of the suppression of the formation of 11-*cis* retinal. Although these trials did not find clear treatment benefits in slowing the progression of AMD, there is continued interest in developing treatments that limit the accumulation of *bis*-retinoids, as they may be playing a role in other diseases, such as Stargardt.^35^^,^^36^

In wild-type mice reared in cyclic-light, there is the potential for RPE lipofuscin to originate from both 11-*cis* and all-*trans* retinal. In the retinas of mice that lack RDH8, following exposure to light, the generation of all-*trans* retinol is markedly reduced^37^ and all-*trans* retinal levels remain elevated much longer than in wild type.^27^^,^^37^^,^^38^ At the same time, lack of RDH8 does not affect the recovery kinetics of 11-*cis* retinal levels.^27^^,^^37^^,^^38^ Compared to wild type mice, *Rdh8^−^^/^^−^* mice accumulate higher levels of A2E and other *bis*-retinoids.^39^ In isolated *Rdh8^−^^/^^−^* rod photoreceptors, most of the all-*trans* retinal generated by light remains un-reduced in the outer segment,^26^ and there is a significant increase in the levels of lipofuscin precursor fluorophores.^40^ Thus, *Rdh8^−^^/^^−^* mice provide a model, in which the contribution of all-*trans* retinal to the formation of lipofuscin and A2E can be maximized. The increased contribution of all-*trans* retinal is evident in the much higher rates of RPE lipofuscin and A2E accumulation, 20.0 ± 2.7 BU/MP/month and 1.7 ± 0.1 pmol/eye/month, respectively, in cyclic-light-reared *Rdh8^−^^/^^−^* mice compared to 9.1 ± 1.9 BU/MP/month and 1.1 ± 0.2 pmol/eye/month in dark-reared *Rdh8^−^^/^^−^* mice.

This quantification of light versus dark accumulation places an upper limit on the potential contribution of all-*trans* retinal in mice with functional RDH8. In rod photoreceptors from wild-type mice, as well as from strains with other defects but which contain functional RDH8, following release from photoactivated rhodopsin, all-*trans* retinal comprises 20% of the total retinoid, the other 80% being all-*trans* retinol.^33^^,^^41^ In the living eye, the all-*trans* retinal levels are further decreased through the action of IRBP, the specialized carrier present in subretinal space; extracellular IRBP lowers outer segment retinoid levels by approximately fourfold.^40^ Taken together, these findings indicate that in rod photoreceptors with functional RDH8, the levels of all-*trans* retinal will be approximately 5% of those in *Rdh8^−^^/^^−^* mice. Yet, despite having relatively high levels of all-*trans* retinal, cyclic-light-reared *Rdh8^−^^/^^−^* mice accumulate lipofuscin and A2E in the RPE at a rate of only approximately two times higher than dark-reared *Rdh8^−^^/^^−^* mice. This difference in the rate of A2E accumulation in the RPE cannot be accounted for by a difference in the rate of rod outer segment phagocytosis, which is broadly similar in cyclic light and in darkness,^42^ and, if anything, somewhat lower in the dark.^43^ Unfortunately, a direct comparison between the rates of A2E accumulation in *Rdh8^−^^/^^−^* and wild type mice is fraught with difficulties, as the rate of A2E accumulation depends on several factors, such as pigmentation, the particular 450Rpe65 variant, or the ambient light intensity during the light cycle. Nonetheless, the available comparisons for 3 to 10 month old cyclic-light-reared animals show that the levels of A2E in *Rdh8^−^^/^^−^* mice are only approximately four times higher than those in wild type (see fig. 5C in ref. 37, fig. 10A in ref. 27, fig. 1C in ref. 38, and fig. 4 in ref. 39). For lipofuscin accumulation, a direct comparison of *Rdh8^−^^/^^−^* with isogenic wild type mice is not available. The available data show that for cyclic-light-reared animals lipofuscin accumulates only approximately 4-times faster in *Rdh8^−^^/^^−^* (20.0 ± 2.7 BU/MP/month) compared to wild type (5.6 ± 0.9 BU/MP/month).^16^ At 1 month of age, lipofuscin levels are similar in *Rdh8^−^^/^^−^* (7.3 ± 2.2 BU/MP) and wild type (10.7 ± 1.2 BU/MP),^16^ and at 6 months approximately 4 times higher in *Rdh8^−^^/^^−^* (112.2 ± 13.1 BU/MP) compared to wild type (36.7 ± 1.8 BU/MP; see Fig. 2).^16^ Therefore, the modest increase in the rate of A2E and lipofuscin accumulation observed in cyclic-light- compared to dark-reared *Rdh8^−^^/^^−^* mice supports the view that the contribution of all-*trans* retinal to the formation of *bis*-retinoids and lipofuscin in wild-type mice is much smaller than that of 11-*cis* retinal.

The reductase activity of RDH8 keeps in check the generation of *bis*-retinoids from all-*trans* retinal released by photoactivated rhodopsin; unsurprisingly, there is no significant increase in lipofuscin precursor fluorescence in wild-type mouse rods following light exposure.^16^ In contrast, as 11-*cis* retinal is not a substrate of RDH8,^30^ addition of exogenous 11-*cis* retinal to isolated wild type mouse rods results in a significant increase in lipofuscin precursor fluorescence.^16^ *Bis*-retinoid formation from excess 11-*cis* retinal is kept in check through an initial step of lipid-catalyzed isomerization to all-*trans* retinal, which can then be reduced to all-*trans* retinol by RDH8 in photoreceptor outer segments.^17^ Exogenous 11-*cis* retinal and all-*trans* retinal entering photoreceptor inner segments are reduced by the dual substrate specificity of the resident enzyme RDH12.^26^^,^^44^ The minimal contribution of all-*trans* retinal to *bis*-retinoid formation suggested by our studies is consistent with the similar rates of RPE lipofuscin and A2E accumulation in cyclic-light- and dark-reared pigmented mice with functional RDH8.^16^^,^^45^ Interestingly, in nonpigmented mice, *bis*-retinoid levels are lower in cyclic-light-reared animals due to photodegradation.^45^

Given the above considerations, it is only in cyclic-light-reared *Rdh8^−^^/^^−^* mice that all-*trans* retinal makes a substantial contribution to RPE lipofuscin. This contribution does not make any substantial difference to the fluorescence emission spectra of lipofuscin granules, which are the same for cyclic-light- and dark-reared *Rdh8^−^^/^^−^* animals (see Fig. 1B). This is to be expected given that reactions of retinaldehyde with outer segment components give rise to essentially the same *bis*-retinoid species, regardless of whether the original retinaldehyde isomer is all-*trans* or 11-*cis*.^17^ In wild type mice, the fluorescence emission spectra of lipofuscin granules are also the same for cyclic-light- and dark-reared animals,^16^ although, in that case, as argued above, most of the content of RPE lipofuscin granules would originate from 11*-cis* retinal.

The levels of A2E found in the retinas of *Rdh8^−^^/^^−^* mice were fairly substantial, 1.9 ± 0.4 and 1.7 ± 0.1 pmol/eye in cyclic-light- and dark-reared animals, respectively, at 6 months of age. These levels are in the order of 20% to 35% of the levels that have accumulated in the RPE. Similarly high levels of A2E have also been found in the retinas of wild type mice.^16^ As it takes only 10 days for the full length of a mouse rod outer segment to be phagocytosed by RPE,^46^^,^^47^ the total amount of A2E entering the RPE in a few month's period would be several times higher than the amount observed accumulating. This would be an indication that A2E, and presumably other *bis*-retinoids as well, is being processed within the RPE. The nature of this processing is as yet unknown, but could involve degradation, expulsion from the RPE, or conversion to nonextractable components to name a few possibilities. Additional evidence for the processing of A2E within the RPE was provided by moving *Rdh8^−^^/^^−^* mice from light to darkness (see Fig. 4). When the A2E levels were determined at 6 months of age, animals that had spent the previous 1 to 8 weeks in darkness had lower A2E levels than estimates indicated they had just before being moved to darkness (see Fig. 4). A2E exhibits a wide range of cytotoxic properties that include the inhibition of a range of cellular functions, such as the processing of phospholipids and cholesterol by RPE lysosomes.^12^^,^^48^ The processing of A2E itself opens the possibility of mitigating its cytotoxicity by promoting its removal. Such an approach would sidestep the deleterious side effects associated with the suppression of the formation of 11-*cis* retinal. It should be noted that the process of A2E accumulation appears to be fairly complex. Although substantial amounts of A2E were found in the retina, they were not significantly different between cyclic-light- and dark-reared animals, 1.9 ± 0.4 and 1.7 ± 0.1 pmol/eye respectively at 6 months of age. The large difference in the RPE A2E levels between cyclic-light- and dark-reared *Rdh8^−^^/^^−^* mice could suggest that a significant amount of A2E forms in the RPE itself, from precursors originating in phagocytosed rod outer segments, such as A2PE,^49^^,^^50^ all-*trans* retinal, as well as 11-*cis* retinal from rhodopsin.^51^ Another possible explanation for the difference in RPE A2E levels would be a lower rate of A2E processing in cyclic light compared to darkness.

In summary, we have measured the accumulation of lipofuscin and A2E in the RPE of cyclic-light- and dark-reared mice that are deficient in RDH8, the enzyme that reduces all-*trans* retinal to all-*trans* retinol in photoreceptor outer segments. The results support the view that in wild-type retinas with functional RDH8, most of the lipofuscin and A2E that accumulate in the RPE originate from reactions of 11-*cis* retinal, both in darkness and in the presence of light. The decrease in A2E accumulation observed in mice moved from light- to dark-rearing is consistent with substantial processing of A2E in the RPE, suggesting the presence of a mechanism that could be targeted to control A2E cytotoxicity.
